# The Enigmatic Emerging Role of the C-Maf Inducing Protein in Cancer

**DOI:** 10.3390/diagnostics11040666

**Published:** 2021-04-08

**Authors:** Mario Ollero, Dil Sahali

**Affiliations:** 1 Univ Paris Est Creteil, INSERM, IMRB, F-94010 Creteil, France; dil.sahali@inserm.fr; 2 AP-HP, Hopital Henri Mondor, Service Néphrologie, F-94010 Créteil, France

**Keywords:** antiangiogenic therapy, solid tumors, idiopathic nephrotic syndrome

## Abstract

The C-Maf-Inducing protein (CMIP) was first described as overexpressed in T cell subpopulations of idiopathic nephrotic syndrome (INS) patients. Later, it was found concomitantly upregulated in podocytes. CMIP expression has also been reported in several types of cancer, including blood malignancies and solid tumors, in many cases accompanied by nephrotic syndrome. In addition to these observations, the duality of CMIP overexpression in the kidney and INS lesions, has been extensively reported as one of the adverse effects of anticancer therapy based on anti-receptor tyrosine kinase drugs. As a consequence, a growing body of evidence points at CMIP as playing a role in cancer. This includes its reciprocal regulatory ties with NF-κB and WT1, and the more recent reports showing an involvement in regulatory circuits in cancer cells. The ensemble of the current information justifies to propose CMIP as an important piece of the puzzle of biological systems involved in cancer and other diseases and its potential as a target.

In this review the enigmatic role of the c-Maf inducing protein in two sides of cancer is addressed. First, we will present the structural features of the protein, the discovery, and the links with pathophysiologic processes. Next, its abnormal expression in different types of cancer, as well as its association with renal toxicity in response to anticancer therapies will be considered. Then, we will examine the known mechanisms involved in the regulation of its expression and will end by discussing the functional relevance in the crossroads between cell death and survival.

## 1. C-Maf Inducing Protein: Structural and Functional Aspects

To investigate the molecular mechanisms responsible for minimal change nephrotic syndrome (MCNS), one of the forms of idiopathic nephrotic syndrome (INS) characterized by a relapse-remission course, our team performed a subtractive cloning and differential screening on T cells isolated from patients [1]. The study resulted in the finding of a differentially expressed gene, catalogued as *Kiaa1694.* The 5’ coding sequence was next isolated and the gene subsequently rebaptized as C-maf inducing protein and given the acronym CMIP [2]. The new attributed name was due to the upregulation of the transcription factor C-maf in Jurkat cells transfected with the *CMIP* coding sequence. 

The original isolation and cloning from brain tissue [3] and the more recent genetic linking studies [4] point at a function of CMIP in the nervous system, while in other tissues its expression in physiological conditions is moderate and in some cases almost negligible. Interestingly, constitutive knockout results in lethality, suggesting a role in early development (unpublished observations). Conversely, the conditional deletion of Cmip in the developing kidney in mice does not induce any alterations in morphology or function, indicating that CMIP has no role in glomerular development and maturation [5]. Overexpression of CMIP has been mostly reported in relation to glomerulopathies. Accordingly, CMIP upregulation in PBMC and in podocytes is associated with relapses of MCNS [6,7], in membranous nephropathy glomeruli [8] and in podocytes in some forms of lupus nephritis [9]. 

CMIP abundance in podocytes is also augmented in rodent models of nephropathy, such as the LPS-induced [7] and Adriamycin-induced [10] mice, and the Heyman nephritis rat model [8]. Likewise, the podocyte specific knock-in of human CMIP in mouse results in the development of proteinuria and MCNS-like lesions, whereas the knockdown of endogenous Cmip prevents proteinuria occurrence in an experimental model of nephrotic proteinuria [7]. Comparison of transcripts shows that mouse *Cmip* exhibits 98.4% and 99.9% homology to the human and rat coding sequences, respectively.

**Figure 1:**
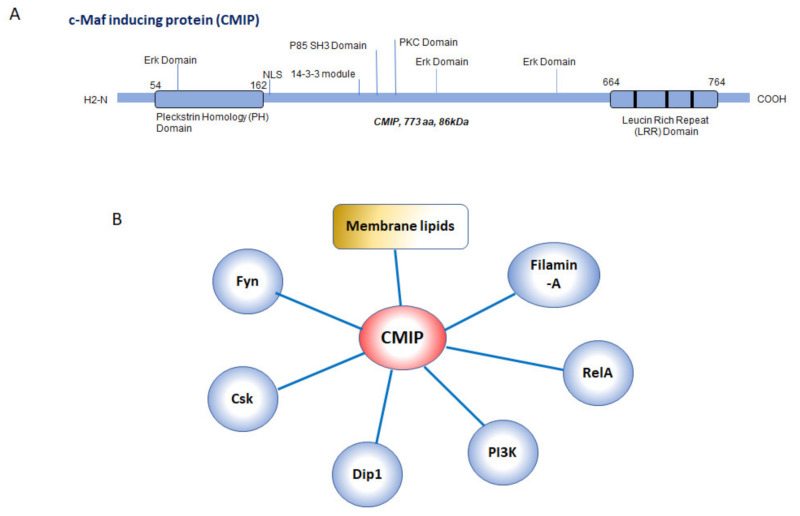
The C-Maf-Inducing protein (CMIP) structure and interactome. (**A**): Predicted structure of CMIP protein. The main domains and modules are indicated (NLS: nuclear localization sequence). (**B**): reported and observed interactions of CMIP.

The *CMIP* gene comprises 21 exons spanning 268 kb in length on chromosome 16q24 [2] and encompassing a coding sequence of 2319 nucleotides that encodes an 86kDa protein. The predicted structure includes an N-terminal pleckstrin homology (PH) domain, a C-terminal leucin rich repeat, some potential phosphorylation sites for protein kinase C (PKC) and casein kinase 2 (CK2), a nuclear localization motif, an Erk domain and an SH3 domain similar to that of the PI3 kinase regulatory unit [11] (Figure 1A). The full protein interacts with membrane lipids via the PH domain in the lipid raft fraction (unpublished results). The latter indicates potential membrane associations, while its observed localization is mostly cytosolic and nuclear [2,7,12]. 

Altogether, the numerous interacting domains predicted suggest a rich interactome and a potential adaptor function (Figure 1B). A handful of protein partners of CMIP have been identified so far by yeast two-hybrid and immunoprecipitation. The first recognized partner was filamin-A, a cytoskeletal protein [12]. Other confirmed interactors are the RelA subunit of the nuclear factor kappa-B (NF-κB) transcription factor [10,11], the p85 regulatory subunit of phosphoinositide-3 kinase (PI3K), and the death activated protein kinase-interacting protein 1 (Dip1) [13]. In the vicinity of lipid rafts, an interaction with the Src kinase Fyn has been described in several cell types [7], within a complex involving the protein associated with glycosphingolipid-enriched microdomains (PAG) [14]. Under angiotensin II stimulating conditions CMIP has been found to interact with the C-terminal Src kinase (Csk) in podocytes [15] (Figure 1B). 

**Figure 2:**
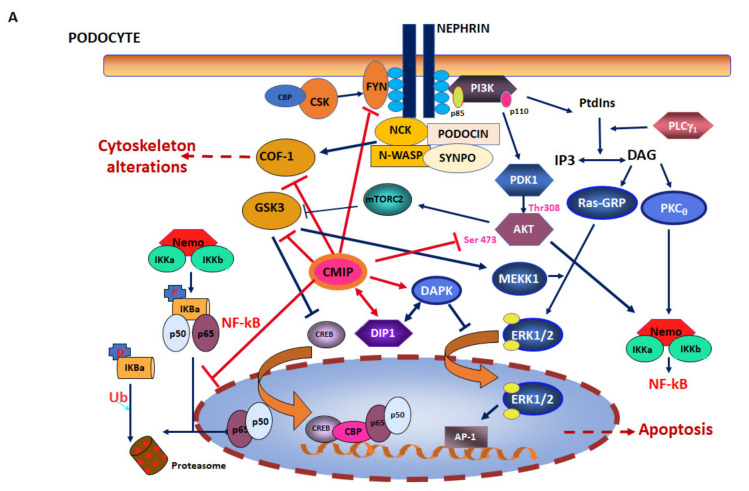

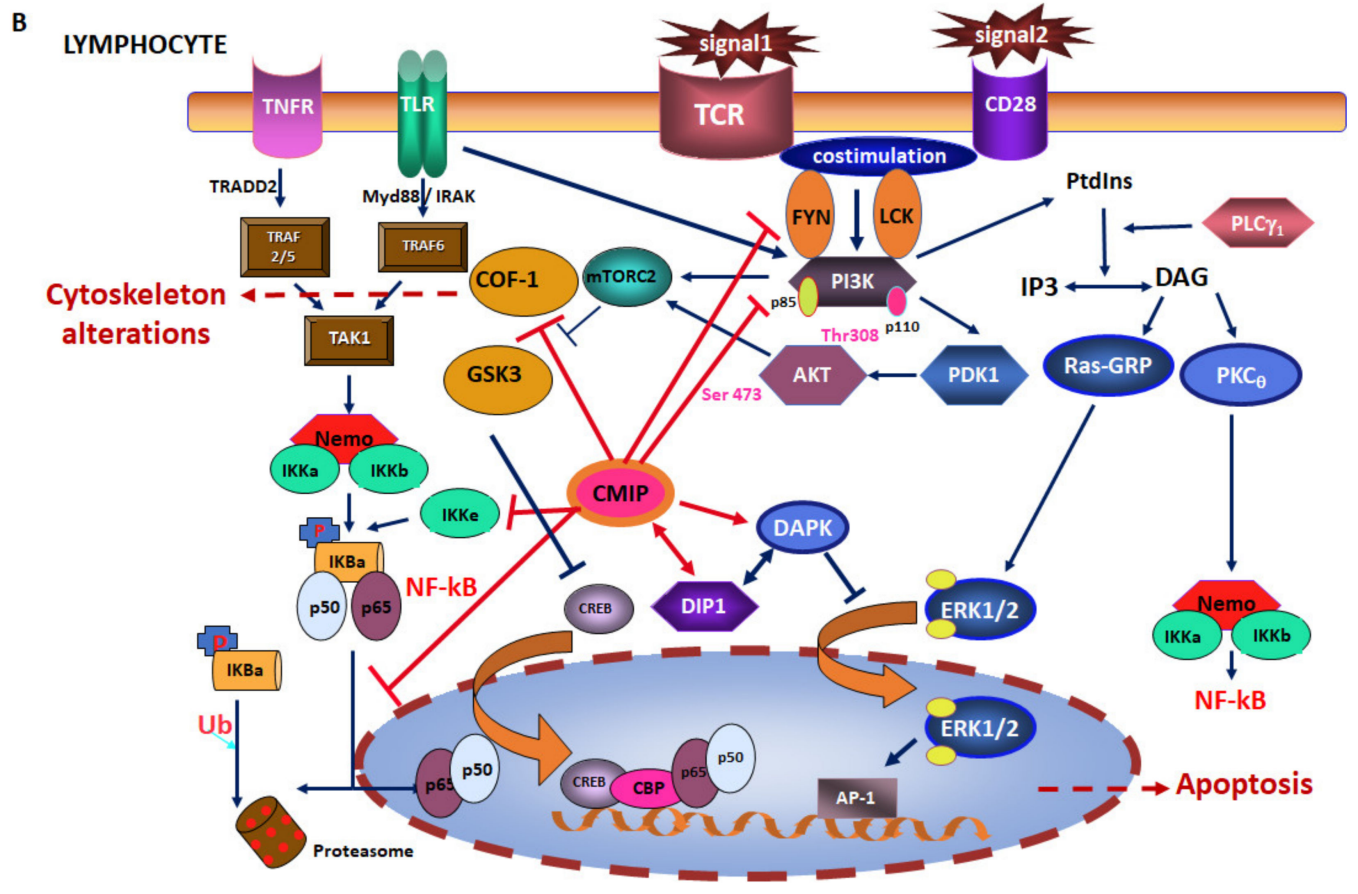
Impact of CMIP expression on signaling pathways. CMIP inhibits several key regulators of main signaling pathways and functions in the cell, these effects indicated by red arrows. (**A**): in the podocyte, CMIP inhibits FYN, resulting in a defect in nephrin phosphorylation. This alters downstream interactions and signaling events involving, among others, cytoskeleton regulators (NCK, N-WASP, podocin) and cytoskeletal proteins (SYNPO), resulting in cytoskeleton disorganization. (**B**): in the lymphocyte, CMIP inhibits signaling events associated with TCR-CD28 co-stimulation, by inactivating Src kinases FYN and LCK. In both the podocyte (**A**) and the lymphocyte (**B**), by inhibiting COF-1, CMIP alters cytoskeleton actin polymerization. By inhibiting PI3K, mTOR (PDK1, AKT, mTORC2), MAP kinase (Ras-GRP, MEKK1, ERK1/2) and NF-κB pathways are negatively impacted. NF-κB, which can be activated by different stimuli, can be inhibited by CMIP in different fashions (inhibition of IKKε expression, inhibition of I-κB phosphorylation, inhibition of I-κB proteasomal degradation). The ensemble of these effects ultimately results in cell apoptosis. (AKT: protein kinase B; AP-1: Jun-Fos transcription factor; CBP: CREB binding protein; COF-1: cofilin-1; CREB: cAMP response element binding protein; DAG: diacylglycerol; ERK: mitogen activated protein kinase; IKK: I-κB kinases; IP3: inositol tris-phosphate; IRAK: interleukin 1 receptor associated kinase; LCK: lymphocyte cell-specific protein-tyrosine kinase; MEKK: mitogen activated protein kinase kinase kinase; mTORC2: mammalian target of rapamycin complex 2; Myd88: innate immune signal transduction adaptor; NCK: Non-catalytic Region of tyrosine kinase; NEMO: IKKγ; N-WASP: Neural Wiscott–Aldrich Syndrome Protein; PDK1: phosphoinositide dependent kinase 1; PKCθ: protein kinase C θ; PLCγ: phospholipase C γ; PtdIns: phosphoinositides; Ras-GRP: RAS guanyl releasing protein; SYNPO: synaptopodin; TAK1: TGFβ activated kinase 1; TLR: toll-like receptor; TNFR: tumor necrosis factor receptor; TRADD: TNFRSF1 associated via death domain; TRAF: TNF receptor associated factor 2; Ub: ubiquitin.)

As stated above, the potential to interact with multiple proteins of diverse functions and with membrane lipids led us to consider CMIP as an adaptor protein. However, elucidation of its ultimate functions is currently underway by means of overexpression and invalidation models. In particular, interactions with RelA, PI3K, and Dip1 place CMIP at the functional crossroads of survival/death pathways, which may be decisive in cell fate, and impact differently normal and cancer cells (Figure 2). 

Experimental work developed mostly by our team has provided invaluable information about the molecular function of CMIP. A role in the regulation of signaling associated with the immune response has been consistently suggested. Thus, expression of the human protein in mouse T cells in vivo inhibits T cell receptor (TCR) clustering and activation. Consequently, transgenic T cells exhibit a lower proliferative capacity and are less prone to producing cytokines after stimulation. In addition, transgenic mice display a higher percentage of T cells with a naïve T-cell phenotype, as well as a lower reactivity to CD3/CD28 stimulation, as reflected by a decrease in IL-2 production [16]. Expression of a truncated form (TCmip) was associated with cell aggregation and abnormal distribution of the cytoskeletal protein L-plastin after anti-CD3/CD28 activation [2]. 

Likewise, the impact of CMIP expression on podocyte signaling and subsequently on cytoskeleton organization is supported experimentally. CMIP has been shown to inactivate the serine-threonine kinase glycogen synthase kinase beta (GSKβ) [11], to inhibit nephrin phosphorylation [10], and protein kinase B (Akt) signaling [7] in podocytes. CMIP has been shown to be involved in the nephrin phosphorylation defect induced by Angiotensin II in podocytes, through inhibition of the Csk-Fyn-Akt axis [15]. Some of the findings related to CMIP interfering with cell signaling pathways have been obtained in both lymphocytes and podocytes. For example, Fyn inhibition has been reported in both cell types [7,15,16]. Likewise, CMIP has been shown to inactivate cofilin-1 (COF-1), thereby interfering with actin polymerization, and altering cytoskeletal structure in podocytes [17], and in lymphocytes [16] (Figure 2).

Apart from their undeniable association with podocyte diseases, changes in CMIP expression have been reported in other cell types and pathophysiologic conditions. Thus, CMIP abundance has been found negatively associated with insulin-stimulated glucose-conversion lipogenesis in abdominal subcutaneous adipocytes [18]. In methylome wide analyses, an increased methylation of CMIP promoter has been associated with HDL cholesterol efflux capacity [19] and with the development of obesity [20]. Other findings include that of CMIP peptides bound to MHC class II molecules isolated from lymph nodes in a mouse model of colitis [21], which suggests that CMIP could be an autoantigen in autoimmune conditions.

Finally, a potential function of CMIP in other organs and tissues can be inferred by the association of genetic variants with a number of pathologies. Accordingly, polymorphisms in CMIP sequence have been linked to the susceptibility to developing type 2 diabetes [22,23,24,25], to a decreased homeostasis model assessment of β-cell function and increased fasting plasma glucose [26,27], to adiponectin levels [28,29], to language impairment and autism [30,31,32,33], to adenylate cyclase 3 deficiency in the olfactory epithelium [34], to dyslipidemia associated with IgA nephropathy [35,36], to blood lipoprotein content [37], and adipocyte lipolysis [38]. Moreover, partial deletions have been described in some patients suffering from autism and gastro-intestinal disorders [39].

In addition to these observations, a growing body of evidence suggests a close functional link between CMIP and cancer.

## 2. CMIP in Cancer Cells

### 2.1. CMIP in Hodgkin Lymphoma

The first link between cancer and INS was established in a subpopulation of Hodgkin lymphoma patients, where it was reported as a paraneoplastic glomerulopathy [40]. Later, in a cohort of Hodgkin patients undergoing MCNS, our team found an increased expression of CMIP in podocytes at the RNA and protein levels, which was accompanied by a strong expression in Hodgkin and Reed Sternberg (HRS) cells in lymph nodes [14]. In HRS, CMIP was found to interact with Fyn and PAG, while its overexpression was associated with decreased Fyn abundance [14] (Table 1).

### 2.2. CMIP in Solid Tumors

CMIP expression has also been found in solid tumor cells. Paraneoplastic nephrotic syndrome, like the one described in the context of Hodgkin lymphoma, has been reported by our team in a patient of small cell lung carcinoma presenting with INS [41]. Concomitant upregulation in both tumor cells and in podocytes suggested the participation of a circulating factor as responsible for the glomerular pathology. In gastric cancer cells, CMIP expression is associated with lower survival, while its depletion by RNA interference leads to decreased cell proliferation and migration [42]. Gastric cancer of the HER2 positive type has shown different responses to treatment with the immunotherapeutic drug Herceptin. Gastric cancer cells resistant to this drug displayed increased CMIP expression, the latter correlated with tumor size and lymph node metastasis by a SOX2-mediated mechanism [43]. Likewise, CMIP expression has been reported in poor prognosis forms of glioma [44] and suggested as a marker differentiating melanoma from nevus [45]. CMIP has also been detected in choriocarcinoma trophoblastic [46] and in colorectal cancer [47] cell lines. In a bioinformatics analysis on data extracted from the Gene Expression Omnibus (GEO) database and the cancer Genome Atlas, aiming to identify prognostic biomarkers of breast cancer, CMIP was included as one of eight hub genes associated with the progression and poor prognosis of breast cancer [48]. As a consequence of these findings, several authors have defined CMIP as oncogenic [42,44] (Table 1).

## 3. CMIP in Antiangiogenic Therapy

A different perspective associates CMIP and cancer, as its expression is specifically increased in podocytes from patients treated with receptor tyrosine kinase inhibitors (RTKI). RTKI constitute one of the antiangiogenic strategies followed as a first line therapy in several cancers. One of these approaches consists of targeting the vascular endothelial growth factor (VEGF) ligand by means of specific antibodies. The second one is to target the receptor activity with RTKI. A proportion of patients following one program or the other develop renal toxicity. We found that, most interestingly, the VEGF-targeting intervention resulted in thrombotic microangiopathy, while the RTKI-based led mainly to nephrotic syndrome of MCNS type [49]. Strikingly, CMIP was overexpressed in biopsies from patients corresponding to the latter condition, strongly suggesting this protein as a marker of RTKI-induced podocytopathy. Another remarkable finding was the significant downregulation of RelA in glomeruli from RTKI-treated patients, conversely to those from anti-VEGF-treated, which showed increased RelA expression and no CMIP detection. This complementary counterbalance of both proteins is a relevant element in CMIP function, in its relation to cancer and is developed below [50].

## 4. Regulation of CMIP Expression: The Dangerous Dance with WT1 and NF-κB

The regulation of CMIP expression is far from being completely understood. In physiological conditions its expression is limited to background levels in most tissues, most likely due to epigenetic mechanisms. Indeed, epigenetic regulation of CMIP expression has been evoked in placental tissues associated with premature birth. CMIP has been identified as one of the genes showing an abnormally low methylation pattern in a genome wide methylome analysis of placental tissues in preeclampsia and term birth as compared to preterm birth [46]. These methylation levels negatively correlate, as expected, with the RNA expression data, indicating lower CMIP expression in preterm birth but indirectly implying relatively moderate levels in normal placental tissues. This observation is reminiscent of the recent finding that constitutive Cmip knockout is lethal in mice (unpublished results) and suggests promoter methylation as a possible general strategy to limit CMIP expression. 

**Figure 3:**
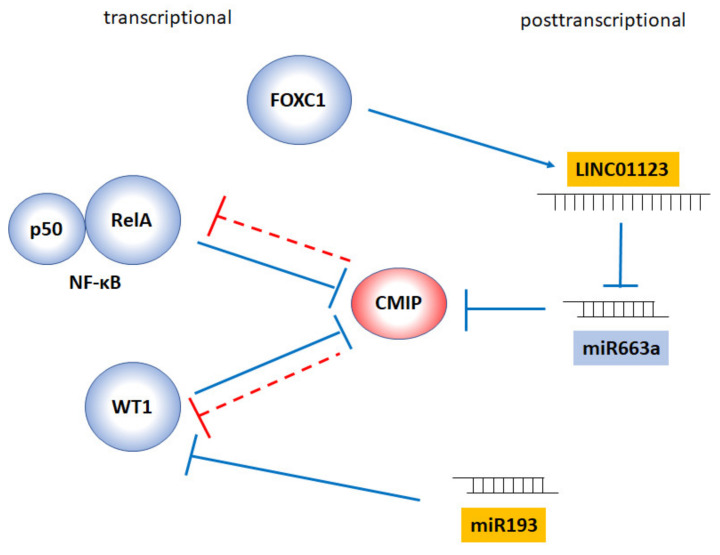
Reported transcriptional and posttranscriptional mechanisms regulating CMIP expression. Those factors inhibiting CMIP expression are shown in blue, and those inducing its expression are in yellow. Direct and indirect effects are represented. Red discontinuous lines indicate the reciprocal inhibitory effects of CMIP on NF-κB and WT1.

Furthermore, post-transcriptional regulatory mechanisms have been described based on the activity of two micro-RNAs, namely, miR-101-3p [42] and miR-663a [51]. From the latter, it is inferred that LINC01123, a long non-coding RNA with a sponging function on miR-663a, is able to increase the stability of the CMIP transcript. This was proven in two lines of human breast cancer cells (MDA-MB-231 and HCC1937). LINC01123 is transcriptionally activated by FOXC1 (Figure 3). 

While no transcriptional activators of CMIP have been defined so far, to date two transcriptional repressors have been identified (Figure 3). One is the Wilms’ tumor suppressor protein (WT1), a master transcription factor for kidney development and homeostasis [52] and a dual actor displaying a tumor suppressor and oncogenic role in normal and malignant hematopoiesis [53]. The other is NF-κB. In physiological conditions, CMIP transcription is inhibited by WT1 [5]. WT1 is expressed selectively in podocytes in the adult kidney, where it activates the transcription of genes encoding key proteins of the slit diaphragm, and its downregulation is characteristic of Denys–Drash and Frasier syndromes in which, conversely, CMIP is upregulated [5]. When WT1 is negatively regulated post-transcriptionally by miR-193, CMIP is also overexpressed in podocytes [54]. Most intriguingly, we have found that CMIP, in turn, induces the proteasomal degradation of this transcription factor, as a result of an E3 ubiquitin ligase activity (manuscript in evaluation).

The second known transcriptional repressor of CMIP is NF-κB. We have demonstrated by chromatin immunoprecipitation the binding of this transcription factor to the κB motif on the proximal CMIP promoter. Moreover, in pathological conditions where RelA (the p65 subunit of NF-κB) is upregulated, such as thrombotic microangiopathy—as shown above—CMIP expression in glomeruli is repressed [49]. 

Intriguingly, and as observed on WT1, CMIP has an inhibitory effect on its own transcriptional repressor, NF-κB. Thus, CMIP binds RelA via its LRR domain and inhibits degradation of I-κB, the NF-κB regulator in the cytoplasm [11]. This was demonstrated in transfected Jurkat cells by means of a reporter gene strategy. Similarly, CMIP binds RelA in cultured podocytes and inhibits its nuclear translocation [10,49]. Our early results also pointed at an inhibitory mechanism independent of I-κB phosphorylation, and most likely involving a block in I-κB degradation [11]. Using proteomics we have observed that overexpression of CMIP in mouse T cells leads to downregulation of I-κB kinase epsilon, which is consistent with NF-κB inhibition by targeting a non-canonical mechanism of I-κB phosphorylation [16]. Phosphorylation of RelA at Ser536 was unaffected by CMIP overexpression in Jurkat cells [11] but inhibited in CMIP-transfected mouse podocytes [49]. In addition, in mouse podocytes stably transfected with the human *CMIP* sequence and in biopsies from MCNS patients, CMIP inhibits RelA expression at the protein, but not at the RNA level [55]. The ensemble of these results suggests that CMIP can inhibit NF-κB by different possible mechanisms. 

Altogether, the negative reciprocal interaction between CMIP and its two repressors, and the fact that both transcription factors are known to play a role in the regulation of cell survival, raises the question of CMIP as also playing a crucial part.

## 5. CMIP as a Matter of Life and Death

The negative regulation of NF-κB by CMIP results in increased classic apoptotic markers—high Bax, low Bcl-2, and high cleaved caspase-3- in podocytes [10,55]. In the same cell type, proapoptotic BAD was increased and Bcl-2 decreased CMIP-dependently after angiotensin-II stimulation [15]. Therefore, CMIP has been reported as holding a proapoptotic function in non-cancer cells by two independent laboratories. In addition, overexpression of CMIP in models of glomerulopathy, such as the Heyman nephritis rat model of membranous nephropathy (MN), and in MN patient biopsies, has been linked with increased death-associated protein kinase (DAPK), an apoptosis-related kinase [8] that is also upregulated in CMIP-transfected HEK cells [11].

In line with the early results obtained by our team and others, in a fructose administration model of kidney injury in rats, miR-193a is overexpressed in the glomerulus, which leads to WT1 downregulation and subsequent increase in CMIP abundance, ultimately leading to low RelA expression and apoptosis. This was assessed by Bcl-2, Bax, and Caspase 3 cleavage analysis. Magnesium isoglycyrrhizinate (MgIG), an antiapoptotic drug used as a cytoprotective agent in fructose-induced apoptosis in hepatocytes, reverts this condition [54]. Globally, these results acknowledge a balance between CMIP and RelA as decisive to the life/death fate of cells.

We have analyzed the changes in the global lipidome of mouse podocytes transfected with CMIP. In this untargeted study, we found a specific and unexpected decrease in gangliosides, and an accumulation of their ceramide precursors, including glycosylceramides and lactosylceramides [16]. Ceramides are known to positively regulate apoptosis by changing the balance between the pro- and anti-apoptotic proteins of the BCL-2 family, by forming macrodomains at the mitochondrial membrane that facilitate pro-apoptotic Bax insertion [56]. A ceramide-dependent mechanism of CMIP-induced apoptosis can be hypothesized based on these observations. It is worth noting that ceramide accumulation induced in mouse model by invalidation of the *Asah1* gene, encoding the acidic ceramidase, leads to podocytopathy and nephrotic syndrome [57]. The latter prompts the question of ceramide accumulation as potentially playing a part in the link between CMIP expression and INS.

The results summarized above suggest a regulatory pathway in which NF-κB would represent the effector molecule, resulting in a balance between cell survival and apoptosis. CMIP would play a central role in this scenario. Its dual relation with its two repressors, WT1 and NF-κB, implies positive feedback loops that lead to an amplification of the effect. Stopping these loops would be crucial in podocyte survival but could exert a totally different impact on cancer cells.

## 6. CMIP in Cancer Cells: Friend or Foe?

As shown above, CMIP has been described as a pro-apoptotic protein in podocytes. However, its increased presence in cancer cells challenges the latter as a universal assumption. In triple negative breast and in gastric cancer cells CMIP is significantly expressed and defined by the authors of these reports as oncogenic [42,51]. Transfection of these cells with a vector encoding CMIP and thereby inducing higher expression levels results in increased colony formation and cell proliferation, while CMIP silencing has an opposite effect. Similar conclusions have been reported in a study on glioma cells, where CMIP expression correlates with low relapse-free survival [44]. Interestingly, in the context of melanoma, CMIP and its regulating lncRNA LINC00518 have been described as a two-gene signature to distinguish cancer from hyperpigmented nevi lesions [45]. The ensemble of these reports strongly suggests a dual scenario for CMIP in cancer and normal cells.

An example of this duality is the impact of CMIP expression on the MAPK signaling pathway. In naive T cells CMIP interacts with PI3K and inhibits Lck, resulting in Erk1/2 and p38 activation, but the translocation of the latter to the nucleus is blocked by the upregulation of DAPK as a result of the interaction with the proapoptotic protein Dip1 [13]. Conversely, in gastric cancer cells CMIP induces the activity of SOX2 [43], upregulates MDM2 and MAPK transcription [42].

Assuming that CMIP exerts pro-apoptotic effects in normal cells, and basically inhibits pro-survival pathways and cell proliferation, why is this proapoptotic protein expressed in tumor cells? One possibility is that *CMIP* sequence is mutated in tumor cells—like tumor suppressors such as WT1 in some malignancies-. Indeed, mutations of CMIP have been reported in colon cancers [47,58]. A thorough analysis of these mutations and their impact on CMIP structure could be crucial to understand the basis of its oncogenic function.

Another issue is the expression level. If CMIP is repressed by transcription factors that are generally upregulated in tumor cells, such as NF-κB, there must be an alternative mechanism leading to its upregulation. This could take place in response to a modification in the epigenetic status. In placental tissues, CMIP expression pattern and promoter methylation levels follow that of bladder cancer associated protein (BLCAP) [46]. BLCAP is a tumor suppressor, and regulator of cell proliferation and stimulator of apoptosis. However, BLCAP expression is reduced in carcinomas [59,60]. The methylation status of the CMIP promoter, on the contrary, could be modified in cancer cells. Other regulatory mechanisms, such as non-coding RNAs, could bypass the repressor effect of NF-κB and be at the origin of CMIP overexpression.

Independently of a potential oncogenic role of CMIP in tumors, a collateral effect of its expression is the induction of nephrotic syndrome. Interestingly, the coding sequence of *CMIP* does not seem to be altered in podocyte diseases associated with cancer [14], in contrast to malignancies—as stated above—where multiple mutations have been identified in the CMIP structure, in the absence of related glomerular disease [47,58]. Paraneoplastic nephropathies strongly suggest that the CMIP protein expressed in Hodgkin lymphoma and in some solid tumors keeps an intact structure, function, and deleterious effect on glomeruli. This raises the point of extrarenal circulating factors responsible for glomerular dysfunction.

## 7. Conclusions and Open Questions

Although the expression of CMIP associated with malignancies has been known almost since its discovery, the new high throughput technologies of expression analysis are revealing an unexpected abundance of the CMIP transcript and protein in solid tumors. CMIP has been defined by independent groups as either tumor suppressor—because of its proapoptotic effects—and as oncogenic. This raises several key questions likely to be addressed by future research:

Are the gene sequence and the protein structure identical in both normal and tumor cells?

If so, is the function the same, as regards to inhibition of key signaling pathways, of activation of classic pro-apoptotic markers, or are the cell localization and the molecular functions different? Is CMIP expression part of a counter-regulatory mechanism in cancer cells? Is its pro-apoptotic effect counterbalanced by pro-survival mechanisms?

How is CMIP expression regulated in cancer cells? Are transcriptional repressors bypassed by epigenetic and post-transcriptional circuits?

CMIP is an undeniable target in paraneoplastic syndromes, and a risk factor of renal toxicity in anti-cancer therapies. However, is there a case for CMIP constituting a target in anti-cancer therapy?

## Figures and Tables

**Table 1:** Reported expression of CMIP in cancer cells.

Type of Cancer	Type of Sample	Paraneoplastic Nephropathy Reported	Reference
Hodgkin Lymphoma	HRS Cells	Yes	[14]
Small Cell Lung Carcinoma	Cancer Tissue	Yes	[41]
Gastric Cancer	Cancer Tissue and Cell Lines (MKN45-HR and NCI-N87-HR)	No	[42,43]
Glioma	Cancer Cell Lines (A172, U251)	No	[44]
Melanoma	Surface Melanocytic Lesions	No	[45]
Choriocarcinoma	Trophoblastic Cell Lines	No	[46]
Colorectal cancer	Cell Lines	No	[47]
Breast Cancer	Cell Line Panel	No	[48]

## Abbreviations

Akt: protein kinase B; AP-1: Jun-Fos transcription factor; BLCAP: bladder cancer associated protein; CBP: CREB binding protein; CK2: casein kinase 2; CMIP: C-maf inducing protein; COF-1: cofilin-1; CREB: cAMP response element binding protein; DAG: diacylglycerol; DAPK: death-associated protein kinase; Dip-1: death activated protein kinase-interacting protein 1; ERK: mitogen activated protein kinases; GEO: Gene Expression Omnibus; GSKβ: glycogen synthase kinase beta; HRS: Hodgkin and Reed Sternberg cells; IKK: I-κB kinases; INS: idiopathic nephrotic syndrome; IP3: inositol tris-phosphate; IRAK: interleukin 1 receptor associated kinase; LCK: lymphocyte cell-specific protein-tyrosine kinase; LRR: leucin rich repeat; MEKK: mitogen activated protein kinase kinase kinase; MgIG: Magnesium isoglycyrrhizinate; MCNS: minimal change nephrotic syndrome; MN: membranous nephropathy; mTORC2: mammalian target of rapamycin complex 2; Myd88: innate immune signal transduction adaptor; NCK: Non-catalytic Region of tyrosine kinase; NEMO: IKKγ; NF-κB: nuclear factor kappa-B; NLS: nuclear localization sequence; N-WASP: Neural Wiscott–Aldrich Syndrome Protein; PAG: protein associated with glycosphingolipid-enriched microdomains; PDK1: phosphoinositide dependent kinase 1; PH: pleckstrin homology; PI3K: phosphoinositide-3 kinase; PKC: protein kinase C; PLCγ: phospholipase C γ; PtdIns: phosphoinositides; Ras-GRP: RAS guanyl releasing protein; RelA: p65 subunit of NF-κB; RTKI: receptor tyrosine kinase inhibitors; SYNPO: synaptopodin; TAK1: TGFβ activated kinase 1; TCR: T cell receptor; TLR: toll-like receptor; TNFR: tumor necrosis factor receptor; TRADD: TNFRSF1 associated via death domain; TRAF: TNF receptor associated factor 2; Ub: ubiquitin; VEGF: vascular endothelial growth factor; WT1: Wilms’ tumor suppressor protein.
